# International approaches to paediatric podiatry curricula: It’s the same, but different

**DOI:** 10.1186/s13047-019-0339-9

**Published:** 2019-05-08

**Authors:** Cylie M. Williams, Chris Nester, Stewart C. Morrison

**Affiliations:** 1 Department of Physiotherapy, Monash University, McMahon’s Road, Frankston, VIC 3199 Australia; 2 Peninsula Health, Allied Health, 4 Hastings Rd, Frankston, VIC 3199 Australia; 3 School of Health and Society, University of Salford, Salford, Manchester M6 6PU UK; 4 School of Health Sciences, University of Brighton, Eastbourne, BN20 7UR UK

**Keywords:** Registration, Education, Student, Undergraduate, Children, Curriculum, Paediatric

## Abstract

**Introduction:**

Pre-registration / entry-level programmes of study provide the core knowledge, skills and abilities required for clinical practice. These programmes are where students are introduced to specialist domains of practice and begin to shape their professional interests. The aim of this research was to describe paediatric curricula within pre-registration and entry level podiatry programmes across comparable universities and offer a contemporary synthesis of international practices.

**Methods:**

An exploratory, cross-sectional, online survey was undertaken across a three-month period. Representatives from podiatry programmes delivering pre-registration or entry level podiatry degrees in which graduates are eligible for Professional and Statutory Body registration within their country (deemed at a Bachelor degree or higher), were invited to participate. The survey was administered online using Online Surveys. Descriptive statistics were used to describe the data due to the exploratory nature of the research question and design.

**Results:**

There were responses from seven (54% of 13) universities in the United Kingdom (UK), nine (100% of nine) universities in Australia and four (50% of eight) of the invited universities external to the UK and Australia (New Zealand, Malta, Ireland, South Africa). There was some variation in curriculum content, but all universities reported to cover ontogeny and developmental milestones and general paediatric orthopaedic conditions. There was further discrepancy with the number of hours dedicated to paediatric podiatry within the curricula (ranging from < 5 h to > 26 h).

**Conclusion:**

The findings from this study highlight some disparity in the delivery of training for students relating to paediatrics. The data suggests that there is a need for international coordination in establishing priorities for the paediatric curricula. This will ensure consistency in baseline knowledge, modes of training, amount and nature of curriculum delivery during undergraduate or entry level podiatry training.

**Electronic supplementary material:**

The online version of this article (10.1186/s13047-019-0339-9) contains supplementary material, which is available to authorized users.

## Introduction

Foot and ankle pain in children is common [1] and timely access to services is essential to mitigate longer term complications. Ensuring that graduate podiatrists have the knowledge and clinical skills to assess, diagnose and deliver evidence-informed healthcare for children with foot problems is an important component of professional practice, or within the multi-professional landscape. This is integral to managing foot and ankle problems of this population. Paediatric presentations in any health profession is generally considered as a defined area of practice [2, 3]. This is due to the need for additional knowledge in growth and development of children and young adults. It has historically been considered as treating children and young adults up to the age of 18, but recent recommendations have extended this to the age of 21 [4]. This field of practice requires a cohesive knowledge base blending an understanding of growth and development, developmental biomechanics, paediatric medicine, ethics of paediatric care and child-centred practice. It is essential that clinicians involved in caring for children with foot and ankle symptoms have a defined theoretical framework underpinning their service along with the appropriate skills and competencies in all dimensions of paediatric assessment and treatment. Pre-registration / entry-level programmes provide the core knowledge, skills and abilities required for paediatric practice [2]. There should be inclusion of core paediatric skills and demonstrated competency in this field of pracitce during their training. The content, depth of teaching exposure and breadth of academic and clinical interaction will shape the degree to which the emerging workforce has the skills needed to manage paediatric foot and ankle complaints in practice. The curriculum content of university programmes is heavily influenced by professional bodies together with the social and political landscapes of each country [5]. Each programme each has its own curriculum structure that influence the content of theories and skills taught in the programme. It follows, that exposure of the future podiatry workforce of paediatric knowledge and skills is likely to vary.

Previous research within United Kingdom (UK) based Occupational Therapy programmes reported that paediatrics constituted less than 5% of the content of university programmes [6]. Course content, including paediatric content of occupational therapy programmes varied between countries, [2, 3]. Many programs of study were consistent in that the nature and quantity of exposure to overarching syllabus to drive competency standards, however there were no specific paediatric related teaching pre-registration syllabus required [7]. This echoes work undertaken in medicine where trainee paediatricians rated their performance in paediatric musculoskeletal assessment as poor [8] and self-rated confidence in paediatric musculoskeletal care, low [3]. There are no similar studies within the podiatry context, but national health policies and trends are driving podiatry curricula and experiential learning toward a focus on high-risk foot care. This might challenge the opportunities for a focus on paediatrics education, since it is perhaps considered less urgent than limb and life threatening, expensive, chronic foot and lower limb problems. Nevertheless, there is increasing evidence of common paediatric foot conditions impacting the quality of life of children [9, 10] and foot and leg complaints being the most common adolescent musculoskeletal presentation to primary care professionals [11]. Ensuring that podiatry curricula supports all dimensions of professional practice, including paediatrics, is necessary to ensure that graduates are ready for the demands (and variety) of modern practice. Furthermore, it is a requirement of Higher Education Institutions to ensure delivery of sufficient paediatric content within undergraduate curricula, and to ensure that students have sufficient knowledge to support their readiness for clinical practice, and effective employment. To this end, there is a clear need to understand more about the content, delivery and relevance of paediatric curricula in pre-registration / entry-level podiatry programmes.

The primary aim of this research was to quantitatively describe paediatric curricula within undergraduate and entry level podiatry programmes across comparable universities and offer a contemporary synthesis of international practice(s).

## Methods

### Design

An exploratory, cross-sectional, international online survey was undertaken across a three-month period from February – May, 2018. Ethical approval was granted from the School of Health Sciences Research Ethics Committee, University of Brighton, UK.

### Survey development

Three academic staff with extensive expertise in podiatry education and research developed the survey; two had specific experience in paediatrics. The survey emerged from a structured discourse around course content knowledge between two Universities (one UK and one Australia). A pilot survey was undertaken with academics from the University of Brighton and Monash University. These academics had curriculum development expertise but did not deliver any paediatric podiatry curriculum. This ensured that language was relevant and each question response was consistent with the survey aim. The final survey resulted from the minor amendments made during the pilot. The final survey comprised of three sections:

- Profile of survey responders and their university
- Paediatric specific education content and method of delivery
- Clinical exposure to paediatric cases and the relevant setting(s)

### Participants

A purposive sampling approach was adopted [12]. We sought to recruit representatives from podiatry programmes delivering undergraduate or entry level podiatry degrees in which graduates are eligible for Professional and Statutory Body registration within their country, and deemed at a Bachelor degree or higher. Podiatry courses were identified from the Health and Care Professions Council (UK based) [13], Podiatry Board of Australia [14] and equivalent professional bodies in New Zealand, Canada, Europe and South Africa. The pragmatic decision was taken to only invite course representatives if they delivered their course content in English, the graduates were considered a podiatrist with a similar scope of practice on graduation (excluding degrees focused on surgical training) and/or course data was available in English.

### Procedure

The survey was administered online using Online Surveys [15]. Participants were provided with an overview of the project via the online information sheet, and consented to participation via the online consent statement (Additional file 1). Heads of departments or programme leads were determined from each university website and/ or public listings on podiatry associations/professional body membership websites. All identified potential participants were individually contacted via email at the same time, by a single author, with a consistent invitation script. Participants were informed of the survey close date, and a reminder email was sent 2–3 three weeks prior to this, should there have been no response from the university. Where the head of department was the primary contact point, they were asked to invite another member of university staff to respond if they were uncertain of how to answer the questions, i.e. the staff member responsible for delivery of paediatric curriculum and / or clinical training. All participants provided informed consent prior to completion of the survey, confirming that they had a role relating to the inclusion criteria, that their course had a substantial paediatric content delivered in English and their programme of study graduated podiatrists eligible for registration within their country.

### Data management and analysis

Raw data were extracted into Microsoft Excel (2016). Where there were multiple responders from one institution answers were combined. If they offered different answers to the same question, the answers of the academic with responsibility for paediatric content delivery were used. This was because they are more likely to have detailed knowledge of the topic. If there was knowledge about paediatric course curriculum, the academic with course responsibility was deemed more likely to be aware of the knowledge breadth. As this study was exploratory, descriptive statistics were used (e.g. percentages, median and interquartile range (IQR)) due to the fixed responses or Likert scale used in the survey. All participant’s qualitative comments on additional course content were reviewed and coded against the content headings.

## Results

There were 27 responses from 20 universities; seven (54% of 13 universities) in the UK, nine (100% of 9 universities) in Australia and four (50% of 8 universities) from other countries that were invited. These countries included universities in New Zealand, Malta, Ireland, South Africa, Canada and Europe.

### Participants

One university had three responses from individual staff: the head of department, the academic responsible for paediatric content delivery and another responsible for clinical supervision. There were five universities where both the head of department and academic with responsibility for paediatric content delivery individually completed the survey. The survey was primarily completed by Head of Department(s) of Podiatry or the course team leader (*n* = 15, 75% of universities). Academics with responsibility for delivery of paediatric content (*n* = 11, 55% of universities), clinical supervisors (*n* = 3, 15% of universities) and paediatric course lecturers/supervisors (*n* = 4, *n* = 20% of universities) also completed the survey. Table 1 describes course delivery.

**Table 1:** Participant characteristics and description of paediatric podiatry subject delivery in different countries

	All n (% of 20)	UK n (% of 7)	Australia n (% of 9)	Other n (% of 4)
Course content delivered by:				
*Academic with no paediatric expertise*	10 (50%)	3 (43%)	6 (67%)	1 (25%)
*Academic with paediatric expertise*	6 (30%)	4 (57%)	1 (11%)	1 (25%)
*Sessional or external lecturer*	4 (20%)	0 (0%)	2 (22%)	2 (50%)
Guest lectures delivered by:				
*No guest lectures*	7 (35%)	5 (56%)	3 (33%)	1 (25%)
*Paediatric physiotherapists*	6 (30%)	1 (14%)	5 (56%)	1 (25%)
*Paediatric orthotists*	5 (25%)	2 (29%)	1 (11%)	1 (25%)
*Paediatric occupational therapists*	5 (25%)	0 (0%)	6 (67%)	0 (0%)
*Medical (including orthopaedics, rheumatology, paediatricians)*	3 (15%)	0 (0%)	3 (33%)	1 (25%)
*Unknown*	0 (0%)	0 (0%)	0 (0%)	1 (25%)
Curriculum updates:				
*Annually reviewed and updated*	17 (85%)	6 (67%)	7 (78%)	4 (100%)
*Responsibility of academic*	3 (15%)	1 (14%)	2 (22%)	0 (0%)
Year students are first introduced to paediatric specific content				
1	5 (25%)	2 (14%)	2 (22%)	1 (25%)
*2*	5 (25%)	3 (43%)	2 (22%)	0 (0%)
*3*	7 (35%)	3 (43%)	4 (44%)	1 (25%)
*4*	2 (10%)	0 (0%)	1 (12%)	1 (25%)
*Unknown*	1 (5%)	0 (0%)	0 (0%)	1 (25%)
Approximate hours of paediatric specific content:				
< 5	1 (3%)	0 (0%)	1 (11%)	0 (0%)
5–10	4 (20%)	4 (58%)	0 (0%)	0 (0%)
11–15	1 (2%)	1 (14%)	0 (0%)	0 (0%)
16–20	0 (0%)	0 (0%)	0 (0%)	0 (0%)
21–25	4 (20%)	1 (14%)	0 (0%)	2 (50%)
> 26	11 (55%)	1 (14%)	8 (89%)	2 (50%)
Content is primarily delivered by:				
Face to face lectures	16 (80%)	6 (86%)	6 (67%)	4 (100%)
Face to face tutorials	3 (15%)	1 (14%)	2 (22%)	0 (0%)
Recorded/online lectures	1 (5%)	0 (0%)	1 (11%)	0 (0%)

### Paediatric specific course content and clinical exposure to paediatric cases

Table 2 outlines the topics covered in the courses and the numbers of universities covering the content as per their response on the survey (Additional file 1). There was a median of 9.5 (IQR 8–10) areas covered by all of the courses. Paediatric radiology was covered the least (*n* = 12, 60%) and all universities covered *Ontogeny and developmental milestones* and *General paediatric orthopaedic conditions content (*E.g. *flat feet, rotational or gait relating to osseous or muscular changes that are not neurological)*.

**Table 2:** Paediatric subject course content

	All	UK	Australia	Other
	n (% of 20)	n (% of 7)	n (% of 9)	n (% of 4)
Paediatric specific consultation:				
*Yes*	18 (90%)	6 (86%)	9 (100%)	3 (75%)
*No*	2 (10%)	1 (14%)	0 (0%)	1 (25%)
Embryology and foetal development:				
*Yes*	17 (85%)	5 (72%)	8 (89%)	4 (100%)
*No*	3 (15%)	2 (28%)	1 (11%)	0 (0%)
Ontogeny and developmental milestones:				
*Yes*	20 (100%)	7 (100%)	9 (100%)	4 (100%)
*No*	0 (0%)	0 (0%)	0 (0%)	0 (0%)
Paediatric specific lower limb biomechanical assessments:				
*Yes*	19 (95%)	6 (86%)	9 (100%)	4 (100%)
*No*	1 (5%)	1 (14%)	0 (%)	0 (0%)
Paediatric specific neurological assessments:				
*Yes*	18 (90%)	5 (72%)	9 (100%)	4 (100%)
*No*	2 (10%)	2 (28%)	0 (0%)	0 (0%)
General paediatric orthopaedic conditions content:				
*Yes*	20 (100%)	7 (100%)	9 (100%)	4 (100%)
*No*	0 (0%)	0 (0%)	0 (0%)	0 (0%)
General paediatric neurological conditions content:				
*Yes*	19 (95%)	6 (86%)	9 (100%)	4 (100%)
*No*	1 (5%)	1 (14%)	0 (0%)	0 (0%)
General paediatric conditions content:				
*Yes*	17 (85%)	5 (72%)	9 (100%)	3 (75%)
*No*	3 (15%)	2 (28%)	0 (0%)	1 (25%)
Paediatric specific interventions:				
*Yes*	19 (95%)	7 (100%)	9 (100%)	3 (75%)
*No*	1 (5%)	0 (0%)	0 (0%)	1 (25%)
Paediatric Radiology:				
*Yes*	12 (60%)	2 (28%)	8 (89%)	2 (50%)
*No*	8 (40%)	5 (72%)	1 (11%)	2 (50%)

Seven (35%) universities had a mandatory clinical rotation specific to paediatrics. Nine (45%) universities had general foot health clinics within the university in which paediatric patients attended, suggesting that some students had some exposure to paediatric patients during training. Two universities did not have any internal clinics. Three (15%) universities had placements for students at clinics in health care settings which only had paediatric patients attending and all students attended as part of their training, while 15 (75%) universities reported that some students will see paediatric patients within general external clinical placement in health care settings that were public or private, but that it was not guaranteed. Seven universities offered private/independent sector placements, however it was unknown if paediatric patients attended these. Five (25%) universities did not offer placements in private practices. Participants also provided comments on the challenges with ensuring paediatric case exposure due to large cohorts of students and constrained placement opportunities. Additional comments were made on the difficulty of benchmarking curriculum in countries where their university was the only one delivering a podiatry degree.

### Assessment and competency

Ten (50%) universities had a paediatric specific assessment form for use when children attended internal University clinics, or for training purposes. One (5%) university required students to see a minimum of 10–20 paediatric patients during training. No other universities had a minimum requirement.

A *Paediatric Specific Exam* was the most common method of assessment reported by 7 (35%) universities. This same assessment method was ranked by 12 (60%) universities in the top five types of assessments to determine competence. A paediatric specific oral exam was the second most common method of assessment (13 (65%) of universities), ranking it their preference for assessment for (30%) universities. Paediatric specific individual assignments (*n* = 13, 65%) and paediatric assessments embedded within general examinations (*n* = 11, 55%) were the other two higher ranked methods of assessment, with fewer universities using group assignments (*n* = 9, 45%). Universities used a median (IQR) of 3 (1, 4) methods to assess of paediatric specific student knowledge, with six universities using only one assessment approach.

Assessment and assessment design varied across countries with only two universities within the UK using more than one assessment method (*n* = 2, 22% of 9) compared to all but one of the Australian universities using three or more methods of assessment (*n* = 8, 89%). There were 11 of the universities (55%) having no compulsory or hurdle requirements for students in this topic area. These responses varied across country groups with 5 (71% of 7) of the UK universities having no compulsory / hurdle requirements, 4 (44% of 9) in Australia universities and 2 (50% of 4) of the other country responses having no compulsory / hurdle requirements in paediatric content.

## Discussion

The findings highlight some consistencies in podiatry course content across different geographical territories, but also inconsistencies with the models of delivery and assessment of student competencies in paediatrics. There was a clear disparity in the hours of paediatric content across programmes. Most Australian universities provided more than 26 h of paediatric teaching, whereas in the UK most universities delivered 5–10 h of content. The data from the survey also identified a disparity between the breadth of topics and the number of hours dedicated to the topics. All universities reported they covered the same content but covering 10 paediatric topics in 5–10 h compared with 26 h naturally raises questions about the depth of content and the capacity for students to acquire knowledge effectively.

Furthermore, academics without clinical expertise in paediatrics delivered content in 50% of the universities. This is not a requirement of teaching; however, it raises questions about the suitability of the methods of delivery and may bring into question the depth of knowledge students are exposed to. It may also challenge the expectations of students’ abilities to synthesise and conceptualise paediatric knowledge and translate this into safe and effective paediatric specific clinical practice. Ensuring that students have confidence in paediatric content is important, as effective clinical performance is underpinned by this. Developing confidence in paediatric assessment has been reported to be a problem in paediatric musculoskeletal MSK assessment in other health professions [3]. The data reported in the survey highlights concerns about the time committed to paediatric education and raises concerns about student readiness for practice and more broadly, workforce and professional development specific to the needs of the paediatric care sector. There is a clear need and opportunity for the profession to respond to this via ongoing validated continuing education or through post-graduate education.

There was no trend in the approaches to assessment of students’ knowledge about paediatrics, nor the determinants of competency. Seven of the responding universities offered a paediatric rotation but overall the consensus was that access to a paediatric caseload could not be guaranteed. The inconsistency with mandatory / hurdle requirements suggest that, in some instances, a student may not need to demonstrate competencies in paediatrics before completion of studies. Only one institution had a minimum requirement for students to assess a minimum of 10 paediatric patients. This highlights a concern about the positioning of paediatric podiatry within programme curricula and greater efforts are needed to provide students with access to paediatric cases. This would ensure an appropriate baseline knowledge, skill development and exposure to content for new graduates across many podiatry programs of study.

This survey did not explore the dimensions of clinical / practice-based learning opportunities in paediatrics and it is acknowledged that these may be very rich opportunities for students to advance knowledge and skill in paediatrics. The challenges with ensuring adequate clinical capacity on pre-registration / entry-level programmes, and the sustainability of clinical placement models are also recognised [16]. The differing programme lengths (2, 3 or 4 years of study) may have impacted on the amount of paediatric content delivered. The programme lengths were not taken into consideration during analysis. It is important for education providers to ensure synergy between taught curricula and ensure this aligns with contemporary practice. This research highlights that more efforts are needed to understand more about paediatric content and delivery along with greater access to clinical opportunities to support the graduate workforce. Countries with more than one university delivering programmes of study may use these results as a benchmark for curriculum development or consider standardising content headings within their courses. Globalisation of healthcare and ensuring an international work-ready profession is key to progression and this survey highlights the need for greater effort to be placed in understanding international curricula.

The findings from the survey must be considered in the context of some limitations. While the authors tested the face validity of the tool with educators, no additional testing was undertaken. There was also a decision made to offer a pre-defined list of curricula content to prompt responses and acknowledge this approach may have impacted on the depth of responses. This meant, there is the potential for bias within tool or for universities to over report on content. This study aimed to understand international practices and, due to the nature of the survey, were not able to quantify detailed content being delivered on programmes, nor the specifics of the paediatric content being evidence based.

## Conclusion

This international survey captured current practices with teaching paediatric podiatry in pre-registration and entry level degree programmes. The data suggests that there is a need for international coordination in establishing priorities for the paediatric curricula. This will ensure consistency in baseline knowledge, modes of training, amount and nature of curriculum delivery during undergraduate or entry level podiatry training.

## Additional file

Additional file 1: Paediatric podiatry: an international survey of curriculum content. (PDF 127 kb)

## Abbreviations

IQR: Interquartile range
UK: United Kingdom
