# Supplementary material for: International approaches to paediatric podiatry curricula: It’s the same, but different
Source: J Foot Ankle Res. 2019 May 8;12:28. doi: 10.1186/s13047-019-0339-9 (PMC6507174; doi:10.1186/s13047-019-0339-9)
Supplement: Supplementary file 1 — Paediatric podiatry: an international survey of curriculum content. (PDF 127 kb) [file 13047_2019_339_MOESM1_ESM.pdf]

# Paediatric podiatry: an international survey of curriculum content

## Paediatric podiatry: an international survey of curriculum content

You are invited to participate in an online survey looking at international perspectives of paediatric podiatry curriculum, staffing and clinical paediatric exposure during undergraduate podiatry training across different countries.

This work is being conducted by:
Dr Cylie Williams - Monash University, Australia - Cylie.Williams@monash.edu
Dr Stewart Morrison - University of Brighton, United Kingdom - S.C.Morrison@brighton.ac.uk
Prof Chris Nester - University of Salford, United Kingdom - C.J.Nester@salford.ac.uk

You are invited to participate in this study. Please read this explanatory statement in full before deciding whether to take part in this research. If you would like further information regarding any aspect of this project, you are encouraged to contact the researchers via the email addresses listed above.

**What does the research involve?**
The aim of this research is to gain an international perspective on paediatric podiatry curriculum, staffing and clinical paediatric exposure during undergraduate podiatry training across different countries. If you consent to participate, you will be asked to complete a short survey which will take you approximately 15 minutes. This survey covers basic information about paediatric education and clinical exposure. During this survey, you will also be invited to share any paediatric specific assessment forms your university clinics may use. There are options to indicate if you are unsure or do not know the answer of any questions. Please share this survey with a colleague if they are better placed to complete this.

**Why were you invited to participate in this research?**
You have been invited to participate in this research through direct email from the

researchers due to your role in podiatry education, as specified on your university website. University representatives are invited to participate if the course they coordinate graduates podiatrists to a regulated professional body, your university course content is delivered in English or has a substantial English speaking component specific to paediatric education, teaching staff have an understanding of English or speak English and the course is equivilant to a Bachelor degree or higher.

**Consenting to participate in the project and withdrawing from the research**
Participation in this research is voluntary. You will be asked to check a box confirming that you consent to participate before entering the survey. You are given the opportunity at the end to leave your email address. Once you have answered the survey, if you have left your email address, you can request your answers are withdrawn within 14 days of the survey closure. After that time, your email will be unlinked from the responses and withdrawal of your information will not be possible. You can stop the survey and exit at any time by simply closing your internet browser.

**Possible benefits and risks to participants**
There are no expected benefits from participation in this research, but your responses will inform future research and translation of paediatric specific podiatry information into curriculum. We anticipate gaining valuable knowledge about how podiatry specific paediatric information is taught around the world. You and your university may benefit from this research during future planning of curriculum. No results will be released or published in a way that will identify individuals or universities. There are no questions within this survey expected to cause emotional distress, embarrassment, or discomfort.

**Confidentiality**
You will be asked to identify your university and your role. Your university and role will only be used during reporting of results as a total number of universities and countries that responded. Similarly, frequencies of roles will also be reported during the collation of results. During analysis, the roles and university will be removed and no linkages made, nor will any individual results presented in any presentations or publications resulting from this research that are identifiable.
You will not be asked any information that may be confidential to your curriculum. You will be invited to share a copy of any internal university clinic paediatric assessment forms used during training, this is optional and if shared, thematic aggregate data will be used and no individual universities identified. If you choose to share this information, it will only be viewed by the research team. Your survey answers will be collected by Bristol Online Surveys where data will be stored in a password protected electronic format. We are not collecting identifying information such as your name or IP address but are collecting your email linked to your answers if you chose to provide it. Therefore, your responses will only

remain anonymous to the research team if you do not provide your email. In subsequent reports or publications, yours answers are confidental, and during analysis, will be anonymized. No one will be able to identify you or your answers, and no one will know whether or not you participated in the study.

**Storage of data**
Once the survey is closed, the data will be downloaded from the online server and kept as a data file on a secure and password protected online server for 10 years. All data that we collect will be anonymised and stored electronically.  Only the research team have access to the data.  All data will be anonymised and stored securely for 10 years in accordance with the Data Protection Act.

**Results**
At the end of the project, all participants who request, will be emailed their individual results and a summary of the findings. It is expected that the results will be published in a peer reviewed journal and results presented at relevant conferences by the research team.

**Further information and Complaints**
This research has been approved through the School of Health Sciences Research Ethics Panel, University of Brighton.   Should you have any concerns about the conduct or complaints about the conduct of the project, you are welcome to contact Dr David Haines, Chair of the School of Health Sciences Research Ethics Panel.

You are asked to consent to participate in this survey.  By clicking on the “Agree” button and proceeding with the survey this indicates that:
- You have read the study information
- You voluntarily agree to participate
- You are the appropriate individual to comment on the paediatric curriculum within your podiatry programme of study
- Your university course content is delivered in English or has a substantial English speaking component specific to paediatric education
-The podiatry degree at my university graduates podiatrists who on graduation belong to a regulated profession in my country
You may print a copy of this consent screen for your records. In order to determine your eligibility to participate, please indicate the following: ✱ *Required*

My university is located in: ✱ *Required*

- ◯ Within the United Kingdom
- ◯ Outside of the United Kingdom

# Unitied Kingdom Universities

My university is: ✱ *Required*

- The Birmingham Metropolitan College of Further and Higher Education
- New College Durham
- The University of Huddersfield
- University of Brighton
- University of Southampton
- University of Plymouth
- University of Northampton
- University of Salford
- University of East London
- Queen Margaret University
- Glasgow Caledonian University
- University of Ulster
- Cardiff Metropolitan University

# Outside of UK Universities

My university is: ✱ *Required*

- Queensland University of Technology
- La Trobe University
- Central Queensland University
- Western Sydney University
- University of Newcastle
- Charles Sturt University
- University of South Australia
- University of Western Australia
- Auckland University of Technology
- University of Johannesburg
- Artevelde University College
- Universite du Quebec
- Other

If you selected Other, please specify:

My role is (please all/any that apply): ✱ *Required*

Please select at least 1 answer(s).

- ☐ Head of Department/Programe Lead or equivilant
- ☐ Academic with responsibility for paediatric content delivery
- ☐ Responsibility for paediatric clinical supervision of students
- ☐ Other (Please specify in relationship to paediatric curriculum)

If you selected Other, please specify:

## The following questions relate to paediatric specific content delivered in lectures, tutorials, or online formats.

Paediatric specific education content at my university is primarily delivered by (please choose all that apply): ✱ *Required*

Please select at least 1 answer(s).

- ☐ Permanent employee lecturers with additional qualifications in paediatric related subjects
- ☐ Sessional or casual employee lecturers with additional qualifications in paediatric related subjects
- ☐ Permanent employee general lecturers who have a paediatric curriculum responsibility but no additional paediatric specific qualifications
- ☐ Sessional or casual employee general lecturers who have a paediatric curriculum responsibility but no additional paediatric specific qualifications
- ☐ Other (Please specify)

If you selected Other, please specify:

Undergraduate students may have paediatric specific guest or multi-professional lectures from which of the follow disciplines: ✱ *Required*

Please select at least 1 answer(s).

- ☐ Paediatric orthotist
- ☐ Paediatric physiotherapist
- ☐ Paediatric occupational therapist
- ☐ No paediatric specific guest lectures
- ☐ I don't know if there are any paediatric specific guess lectures
- ☐ Paediatric medical specialisations or other (please specify which specialisation or profession)

If you selected Other, please specify:

How often is paediatric specific curriculum updated? ✱ *Required*

- ◯ Curriculum is annually reviewed and updated as needed
- ◯ Curriculum is biannually reviewed and updated as needed
- ◯ Curriculum review is the responsibility of the lecturer in charge of the subject and I'm unsure when it is updated
- ◯ Other (Please specify)

If you selected Other, please specify:

What year are students first introduced to paediatric podiatry content (E.g. A lecture or tutorial that is solely related to paediatric content)? ✱ *Required*

- First year
- Second year
- Third year
- Fourth year
- Unknown

Approximately how many hours of paediatric specific lectures, tutorials or online learning modules are students exposed to throughout their entire training (E.g. Content solely relates to paediatric assessment, conditions etc.) ✱ *Required*

- < 5 hours
- 5-10 hours
- 11-15 hours
- 16-20 hours
- 21-25 hours
- >26 hours
- Unknown

Students get the majority (the greatest number of hours) of paediatric education content the following way: ✱ *Required*

- Face to face lectures
- Recorded lectures/online lectures
- Face to face tutorials
- Self directed learning with allocated readings

- Unknown

During training, students are exposed to the following paediatric content as part of their (theoretical) learning: ✱ *Required*

Please select at least 1 answer(s).

- [ ] Paediatric specific consultation (this may include room set up, communication skills etc)
- [ ] Embryology and foetal development
- [ ] Ontogeny and developmental milestones
- [ ] Paediatric specific lower limb biomechanical assessments
- [ ] Paediatric specific neurological assessments
- [ ] General paediatric orthopaedic conditions content (E.g. flat feet, rotational or gait relating to osseous or muscular changes that are not neurological)
- [ ] General paediatric neurological conditions content (E.g. Cerebral Palsy, Muscular Dystrophy, Charcot Marie Tooth Disease
- [ ] General paediatric conditions content (E.g. Juvenile idiopathic arthritis, learning disabilities, autism spectrum disorders)
- [ ] Paediatric specific interventions including stretching, strengthening, orthotics, footwear etc
- [ ] Paediatric Radiology
- [ ] Unknown
- [ ] Other (Please specify)

If you selected Other, please specify:

## The follow questions relate to clinical exposure to assessment and treatment of paediatric patients either within a university clinic or on placement.

Do students have a clinical rotation within a paediatric specific university clinic? ✱ *Required*

- Yes, all students are rotated through this clinic as part of their training
- Yes, but not all students rotated through this clinic during their training
- No, our university does not have a paediatric specific university clinics but children attend the general clinics
- No, our university does not have paediatric patients attending the university clinics
- No, our university does not have internal clinics, all hours of experience are attained at external placements
- Unknown

Do students have a clinical placement (either in a hospital or community health service) that is either paediatric specific or have paediatric patients attend? ✱ *Required*

- Yes, all students will have a hospital or community health placement where they will assess or treat paediatric patients
- Yes, but not all students will have a hospital or community health placement where they will assess or treat paediatric patients
- No, our students do not have placements at hospitals or community health services that have paediatric patients
- I don't know if the students see paediatric patients on hospital or community health service placements

Do students have a clinical placement in a private practice that is either paediatric specific or has paediatric patients attending ✱ *Required*

- Yes, all students will have a private practice placement where they will assess or treat paediatric patients
- Yes, all student have a private practice placement, however it is unknown if they see paediatric patients during this
- Yes, but not all students will have a private practice placement where they will assess or treat paediatric patients
- Yes, but not all students will have a private practice placement and it is unknown if

they will see paediatric placements during this

- No, our students do not have placements in private practices
- Unknown

Does your university clinic supervisors have paediatric specific additional training, qualifications or skills? ✱ *Required*

- Yes (please specify training, qualification or skills of any supervisors)
- No
- Unknown

Does your university clinic have a paediatric specific assessment form? ✱ *Required*

- Yes, and I'm willing to share this with the researchers (please email this form to cylie.williams@monash.edu)
- Yes, but I'm not willing/unable to share this with researchers
- No, our clinic does not have a paediatric specific assessment form

During undergraduate training, the minimum number of paediatric patients that must be seen to demonstrate competency is: ✱ *Required*

- >21
- 10-20
- <10
- No minimum number

During undergraduate training, students demonstrate paediatric competency through (please rank from highest to lowest weighting of marks and leave blank through with no marks. You can only select one per line).

Please don't select more than 1 answer(s) per row.

| | 1st (Highest weighting) | 2nd | 3rd | 4th | 5th (Lowest weighting) |
|---|---|---|---|---|---|
| Paediatric specific exam | ☐ | ☐ | ☐ | ☐ | ☐ |
| Paediatric questions embedded within a generalised exam | ☐ | ☐ | ☐ | ☐ | ☐ |
| Paediatric specific individual assignment | ☐ | ☐ | ☐ | ☐ | ☐ |
| Paediatric group assignment | ☐ | ☐ | ☐ | ☐ | ☐ |
| Paediatric specific VIVA or OSCE assessment | ☐ | ☐ | ☐ | ☐ | ☐ |

Which, of any, of the following assessments are hurdle requirements? (Hurdle meaning that if the student fails the particular component, they will not pass the subject or are unable to graduate). ✱ *Required*

- ☐ Paediatric specific examination
- ☐ Paediatric specific individual assignment
- ☐ Paediatric specific OSCE or VIVA question
- ☐ None, students are able to fail the paediatric specific component of any graded work but still pass the subject overall or degree
- ☐ No specific paediatric assessment as part of any hurdle requirements

Do you have any further comments about paediatric undergraduate curriculum that you feel was not covered within this survey?

Thank you for your time. We are also seeking interest for participation in a future Delphi panel to develop the expected undergraduate standards or knowledge requiremet for a new graduate paediatric podiatrists. If you are interested in this or the results from this survey, please indicate below *Optional*

- ◯ Yes, please email me a copy of my answers and summary of the results. I'm interested in being invited to take part in future results.
- ◯ Yes, please email me a copy of my answers and the summary of results only

Please leave your email if you wish for further contact as specified.

Thank you for your time. Please remember, if you indicated an interest in emailing your paediatric assessment form/s or have any questions about participation in this research, please email: cylie.williams@monash.edu

Thank you for your response. At this time, you do not meet the eligibility for participation. If you have any questions about this research, please contact cylie.williams@monash.edu.

# Please close survey window

## Key for selection options

**1 - You are invited to participate in an online survey looking at international perspectives of paediatric podiatry curriculum, staffing and clinical paediatric exposure during undergraduate podiatry training across different countries.**

**This work is being conducted by:**
**Dr Cylie Williams - Monash University, Australia - Cylie.Williams@monash.edu**
**Dr Stewart Morrison - University of Brighton, United Kingdom - S.C.Morrison@brighton.ac.uk**
**Prof Chris Nester - University of Salford, United Kingdom - C.J.Nester@salford.ac.uk**

**You are invited to participate in this study.  Please read this explanatory statement in full before deciding whether to take part in this research. If you would like further information regarding any aspect of this project, you are encouraged to contact the researchers via the email addresses listed above.**

**What does the research involve?**
**The aim of this research is to gain an international perspective on paediatric podiatry curriculum, staffing and clinical paediatric exposure during undergraduate podiatry training across different countries.    If you consent to participate, you will be asked to complete a short survey which will take you approximately 15 minutes. This survey covers basic information about paediatric education and clinical exposure. During this survey, you will also be invited to share any paediatric specific assessment forms your university clinics may use. There are options to indicate if you are unsure or do not know the answer of any questions. Please share this survey with a colleague if they are better placed to complete this.**

**Why were you invited to participate in this research?**
**You have been invited to participate in this research through direct email from the researchers due to your role in podiatry education, as specified on your university website. University representatives are invited to participate if the course they coordinate graduates podiatrists to a regulated professional body, your university course content is delivered in English or has a substantial English speaking**

component specific to paediatric education, teaching staff have an understanding of English or speak English and the course is equivilant to a Bachelor degree or higher.

**Consenting to participate in the project and withdrawing from the research**
Participation in this research is voluntary. You will be asked to check a box confirming that you consent to participate before entering the survey. You are given the opportunity at the end to leave your email address. Once you have answered the survey, if you have left your email address, you can request your answers are withdrawn within 14 days of the survey closure. After that time, your email will be unlinked from the responses and withdrawal of your information will not be possible. You can stop the survey and exit at any time by simply closing your internet browser.

**Possible benefits and risks to participants**
There are no expected benefits from participation in this research, but your responses will inform future research and translation of paediatric specific podiatry information into curriculum.  We anticipate gaining valuable knowledge about how podiatry specific paediatric information is taught around the world. You and your university may benefit from this research during future planning of curriculum. No results will be released or published in a way that will identify individuals or universities. There are no questions within this survey expected to cause emotional distress, embarrassment, or discomfort.

**Confidentiality**
You will be asked to identify your university and your role. Your university and role will only be used during reporting of results as a total number of universities and countries that responded. Similarly, frequencies of roles will also be reported during the collation of results. During analysis, the roles and university will be removed and no linkages made, nor will any individual results presented in any presentations or publications resulting from this research that are identifiable.
You will not be asked any information that may be confidential to your curriculum. You will be invited to share a copy of any internal university clinic paediatric assessment forms used during training, this is optional and if shared, thematic aggregate data will be used and no individual universities identified. If you choose to share this information, it will only be viewed by the research team.   Your survey answers will be collected by Bristol Online Surveys where data will be stored in a password protected electronic format. We are not collecting identifying information such as your name or IP address but are collecting your email linked to your answers if you chose to provide it. Therefore, your responses will only remain

**anonymous to the research team if you do not provide your email. In subsequent reports or publications, yours answers are confidental, and during analysis, will be anonymized. No one will be able to identify you or your answers, and no one will know whether or not you participated in the study.**

**Storage of data**
**Once the survey is closed, the data will be downloaded from the online server and kept as a data file on a secure and password protected online server for 10 years. All data that we collect will be anonymised and stored electronically. Only the research team have access to the data. All data will be anonymised and stored securely for 10 years in accordance with the Data Protection Act.**

**Results**
**At the end of the project, all participants who request, will be emailed their individual results and a summary of the findings. It is expected that the results will be published in a peer reviewed journal and results presented at relevant conferences by the research team.**

**Further information and Complaints**
**This research has been approved through the School of Health Sciences Research Ethics Panel, University of Brighton. Should you have any concerns about the conduct or complaints about the conduct of the project, you are welcome to contact Dr David Haines, Chair of the School of Health Sciences Research Ethics Panel.**
**You are asked to consent to participate in this survey. By clicking on the “Agree” button and proceeding with the survey this indicates that:**
**- You have read the study information**
**- You voluntarily agree to participate**
**- You are the appropriate individual to comment on the paediatric curriculum within your podiatry programme of study**
**- Your university course content is delivered in English or has a substantial English speaking component specific to paediatric education**
**-The podiatry degree at my university graduates podiatrists who on graduation belong to a regulated profession in my country**
**You may print a copy of this consent screen for your records. In order to determine your eligibility to participate, please indicate the following:**

I agree to participate and meet all of the above criteria

I agree to participate but there is one or more of the other criteria that do not apply to me or my university
